# Sub-optimal Specificity of Modified Ziehl-Neelsen Staining for Quick Identification of Tuberculous Meningitis

**DOI:** 10.3389/fmicb.2016.02096

**Published:** 2016-12-27

**Authors:** Ting Wang, Guo-Dong Feng, Yu Pang, Yi-Ning Yang, Wen Dai, Lin Zhang, Lin-Fu Zhou, Jia-Lei Yang, Li-Ping Zhan, Ben J. Marais, Yan-Lin Zhao, Gang Zhao

**Affiliations:** ^1^Department of Neurology, Xijing Hospital, The Fourth Military Medical UniversityXi'an, China; ^2^Department of Neurology, Kunming Medical University affiliated Yan'an HospitalKunming, China; ^3^Department of Neurology, Zhongshan Hospital, Fudan UniversityHaishang, China; ^4^National Center for Tuberculosis Control and Prevention, Chinese Center for Disease Control and PreventionBeijing, China; ^5^Department of Neurology, The General Hospital of the People's Liberation Army Rocket ForceBeijing, China; ^6^The Children's Hospital at Westmead and The Marie Bashir Institute for Infectious Diseases and Biosecurity, University of SydneySydney, NSW, Australia

**Keywords:** tuberculosis meningitis, TBM, Ziehl-Neelsen stain, Xpert MTB/RIF, specificity

## Abstract

**Background:** Microbiological confirmation of tuberculous meningitis (TBM) remains problematic. We assessed the diagnostic performance of a modified Ziehl-Neelsen (MZN) staining method that showed promise in earlier studies.

**Methods:** Patients evaluated for TBM in Shaanxi province, China, were prospectively enrolled from May, 2011 to April, 2013. Cerebrospinal fluid (CSF) specimens were evaluated using the Xpert MTB/RIF® assay, MZN staining, and standard biochemical and microbiological tests, together with detailed clinical and radiological assessment.

**Results:** Among 316 patients included in the study, 38 had definite TBM, 66 probable TBM, 163 possible TBM and 49 “no TBM,” using consensus uniform research case definition criteria. Comparing “definite or probable TBM” to “no TBM” MZN staining had higher sensitivity than Xpert MTB/RIF® (88.5 vs. 36.5%), but greatly reduced specificity (71.4 vs. 100.0%); 14/49 (28.6%) cases with “no TBM” tested positive on MZN. *Mycobacterium tuberculosis* culture was performed in 104/179 (58.1%) of MZN positive samples; 12.5% (13/104) were positive. Using Xpert MTB/RIF® as the reference standard, MZN had a sensitivity of 92.1% (95% CI 79.2–97.3) and specificity of 71.4% (95% CI 57.6–82.2).

**Conclusion:** Xpert MTB/RIF® offered a rapid and specific TBM diagnosis, but sensitivity was poor. MZN was mainly hampered by false positives. Strategies to enhance the sensitivity of Xpert MTB/RIF® or improve the diagnostic accuracy of MZN should be explored.

## Introduction

Tuberculous meningitis (TBM) represents roughly 1% of all cases of tuberculosis (TB), but it is responsible for a disproportionate burden of TB-associated deaths and disability (Thwaites et al., 2013). Diagnosis of TBM is challenging due to atypical early clinical manifestations and the low sensitivity of smear microscopy on cerebrospinal fluid (CSF) specimens. CSF culture is more sensitive, but takes weeks to yield results and requires sophisticated laboratory facilities (Koh et al., 2012). Because of certain death from untreated TBM, a fast and reliable diagnostic test for TBM is urgently needed.

The Xpert MTB/RIF® assay uses real-time PCR to identify pulmonary tuberculosis with a sensitivity of nearly 100% for smear-positive sputum specimens, around 70% for smear-negative (culture-positive) specimens, and near 100% specificity (Nicol et al., 2011). Due to its enhanced sensitivity and ability to simultaneously detect rifampicin resistance, the World Health Organization (WHO) recommended that Xpert MTB/RIF® replace sputum smear microscopy for initial diagnosis of suspected human immunodeficiency virus (HIV)-associated TB (World Health Organization, 2010) WHO also suggested that Xpert MTB/RIF® should be considered as the initial diagnostic test in CSF specimens from patients suspected of having TBM (World Health Organization, 2013), given its specificity and rapid turn-around time compared to *Mycobacterium tuberculosis* culture. A study from South Africa found that CSF Xpert MTB/RIF® detected 67% of culture-confirmed TBM cases; 87% were HIV-infected (Patel et al., 2013). In Vietnam, Nhu and colleagues reported a sensitivity of 59% (specificity 99%) for CSF Xpert MTB/RIF® compared to a clinical TBM case definition; 20.8% of patients were known to be HIV infected (Nhu et al., 2014). WHO recommends CSF Xpert MTB/RIF® as an initial TBM diagnostic test (World Health Organization, 2013) based primarily on two studies that reported sensitivities of 50–60% compared to *M. tuberculosis* culture in patient cohorts with high rates of HIV coinfection (Patel et al., 2013; Nhu et al., 2014). A subsequent study demonstrated that TBM diagnostic sensitivity could be improved using centrifuged CSF (Bahr et al., 2015), but there is limited data about the sensitivity of Xpert MTB/RIF® in HIV uninfected patients.

Modified Ziehl-Neelsen staining (MZN) showed great promise to assist the quick identification of TBM cases. Initial proof-of-concept studies demonstrated greatly improved detection rates, of both extracellular and intracellular mycobacteria, compared to conventional ZN staining (Chen et al., 2012). In a clinical study conducted in China the sensitivity of MZN (82.9%) was found to be significantly higher than conventional ZN staining (3.3%), which had very poor sensitivity (Feng et al., 2014). Since *M. tuberculosis* is an intracellular pathogen and the numbers of extra-cellular bacteria are few in CSF, the ability to stain intracellular and extracellular bacteria was considered the most likely explanation for the increased sensitivity observed with MZN (Thwaites, 2013). However, more rigorous assessment is required to confirm the diagnostic value of MZN and to assess its performance compared to Xpert MTB/RIF® for rapid TBM diagnosis.

## Methods

### Study design and setting

We performed a prospective descriptive study enrolling all suspected TBM patients over a 2 year period (May 2011–April 2013). The study was coordinated from Xijing hospital of the Fourth Military Medical University; the largest medical center in Shaanxi province, China. All tertiary referral hospitals in Shaanxi province (11 in total) participated in the study. Shaanxi province is located in Northwestern China and has a population of 36.7 million people, a TB notification rate of 109/100,000 population (Zhang et al., 2011)^1^ and a low human immunodeficiency virus (HIV) prevalence (< 1% of TB patients) (Zhao et al., 2014). Patients were eligible for inclusion if they had symptoms of meningitis (fever, headache, seizure, vomiting, nuchal rigidity, or abnormal CSF parameters) and provided written informed consent to participate.

### Clinical data

Patients were retrospectively scored according to the British Medical Research Council TBM severity grade (British Medical Research Council, 1948) and classified according to consensus uniform research case definition criteria (Marais et al., 2010), as “definite”, “probable,” or “possible” TBM (Table 1). Since the study aimed to evaluate the diagnostic performance of MZN, MZN was not included in the classification. The “not TBM” category was sub-divided into cases of infectious neurological disorders (IND) and non-infectious neurological disorders (NIND). All patients were tested for HIV (Roche Elecsys HIV Combi, Basel, Switzerland). Additional information was gathered from reviewing relevant clinical notes. Patients were excluded if their clinical data were incomplete or consent for participation was not obtained.

**Table 1:** **Diagnostic criteria and scores assigned by the uniform TBM research case definition (Marais et al., 2010)**.

**Diagnostic criteria**	**Score**
**CLINICAL (MAXIMUM CATEGORY SCORE** = **6)**	
Symptom duration of more than 5 days	4
Systemic symptoms suggestive of TB (1 or more of): weight loss/(poor weight gain in children), night sweats or persistent cough >2 weeks	2
History of recent close contact with an individual with pulmonary TB or a positive TST/IGRA in a child < 10 years	2
Focal neurological deficit (excluding cranial nerve palsies)	1
Cranial nerve palsy	1
**CSF (MAXIMUM CATEGORY SCORE** = **4)**	
Clear appearance	1
Cells: 10–500 per μl	1
Lymphocytic predominance (>50%)	1
Protein concentration greater than 1 g/L	1
CSF to plasma glucose ratio of less than 50% or an absolute CSF glucose concentration less than 2.2 mmol/L	1
**CEREBRAL IMAGING (CT AND/OR MRI) (MAXIMUM CATEGORY SCORE** = **6)**	
Hydrocephalus	1
Basal meningeal enhancement	2
Tuberculoma	2
Infarct	1
Pre-contrast basal hyperdensity	2
**EXTRANEURAL TB (MAXIMUM CATEGORY SCORE** = **4)**	
Chest radiograph suggestive of active TB (excludes miliary TB)	2
Chest radiograph suggestive of miliary TB	4
CT/MRI/US evidence of TB outside the CNS	2
AFB identified or *M. tuberculosis* cultured from another source i.e., sputum, lymph node, gastric washing, urine, and blood culture	4
**Exclusion of alternative diagnoses**- An alternative diagnosis must be confirmed microbiologically, serologically or histopathologically	
**Definite TBM** = AFB seen on CSF microscopy, positive CSF *M. tuberculosis* culture, or positive CSF *M. tuberculosis* commercial NAAT in the setting of symptoms/signs suggestive of meningitis; or AFB seen in the context of histological changes consistent with TB brain or spinal cord together with suggestive symptoms/signs and CSF changes, or visible meningitis (on autopsy).	
**Probable TBM** = Total score of ≥12 if neuroimaging available (total score of ≥10 if unavailable)	
**Possible TBM** = Total score of 6–11 if neuroimaging available (total score of 6–9 if unavailable)	
**Not TBM** = Alternative diagnosis established, without a definitive diagnosis of tuberculous meningitis or other convincing signs of dual disease.	

*TBM, tuberculous meningitis; TB, tuberculosis; TST, tuberculin skin test; IGRA, interferon gamma-release assay; CSF, cerebrospinal fluid; CT, computed tomography; MRI, magnetic resonance imaging; CNS, Central Nervous System; US, ultrasound; AFB, acid-fast bacilli; NAAT, nucleic acid amplification test*.

### Specimen collection and processing

All patients received a lumbar puncture for CSF collection; standard biochemical and microbiological analyses were performed, including India ink staining. CSF *M. tuberculosis* culture was performed using the MGIT960 system according to standard protocol (Krüüner et al., 2006), depending on availability and the discretion of the treating physician. CSF specimens were sent to Xijing Hospital where MZN was performed by experienced technicians, blinded to all other information. MZN staining used 0.5 ml of CSF loaded into a cytospin chamber with poly-lysine-coated slides and centrifuged at 1000 × g for 5 min. The slide was fixed with 4% paraformaldehyde for 15 min at room temperature then permeabilized with 0.3% TritonX-100 for 30 min, before staining with carbolfuchsin containing 0.3% TritonX-100 and counterstained with methylene blue. All slides stained by the modified method were observed under oil immersion at a magnification of 1000 (Chen et al., 2012). Three hundred fields on each slide were examined documenting the number of fields in which acid fast bacilli were observed, as well as their intra- or extra-cellular location. Positive slides were confirmed by independent review from another technician; 25% of slides were randomly selected for rereading (Feng et al., 2014).

All remaining CSF was stored at −80°C (at most 2 years) and then transferred on dry ice to the National Tuberculosis Reference Laboratory of the Chinese Center for Disease Control and Prevention in Beijing to perform Xpert MTB/RIF® (Cepheid, Sunnyvale, CA, USA) testing using centrifugation (1000 rpm for 10 min) of CSF volumes (median: 1 mL, range: 1–2 mL), according to standard protocol (Boehme et al., 2010). All tests were performed by experienced technicians who were blinded to the patients' diagnostic results.

### Statistical analysis and ethics

Statistical analysis was performed using SPSS v.15.0 (SPSS Inc., Chicago, IL, USA) and GraphPad Prism v.5.0 (GraphPad Software Inc., La Jolla, CA). Categorical variables were compared using X^2^ test if they were independent or McNemar's test if they were paired. Differences with *p*-values < 0.05 were considered statistically significant. The study protocol was approved by the Ethics Committee of Xijing Hospital (Fourth Military Medical University—study number KY20105255-1). Written informed consent was obtained from all participants or their legal guardians.

## Results

**Figure 1:**
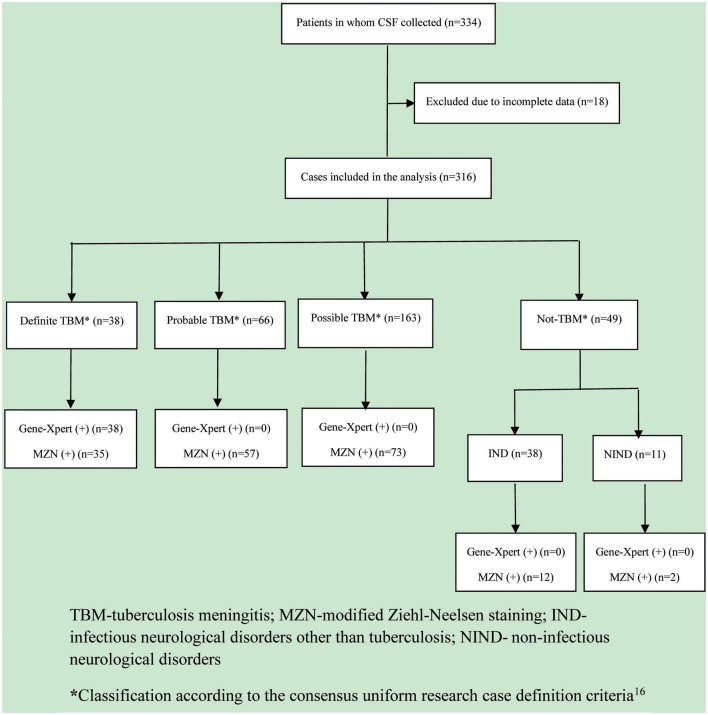
**Flow diagram of patients evaluated for tuberculous meningitis**. TBM, tuberculosis meningitis; MZN, modified Ziehl-Neelsen staining; IND, infectious neurological disorder other than tuberculosis; NIND, non-infectious neurological disorder. ^*^Classification according to the consensus uniform research case definition criteria (Marais et al., 2010).

In total, 334 patients were included in the study, but 18 patients had to be excluded due to incomplete data (*n* = 10) or inadequate consent (*n* = 8). (Figure 1) Of the 316 patients included in the analysis, 38 (12.0%) were classified as definite TBM, 66 (20.9%) as probable TBM, and 163 (51.6%) as possible TBM. Of the 49 (15.5%) cases with an alternative diagnosis that were classified as “not TBM,” 38 (77.6%) had an infectious and 11 (22.4%) a non-infectious cause. The infectious disease (IND) group included bacterial meningitis (*n* = 11), cryptococcal meningitis (*n* = 18), and 9 cases of aseptic meningitis. The non-infectious disease (NIND) group included meningioma (*n* = 3), Guillain-Barre syndrome (*n* = 2), cerebral hemorrhage (*n* = 2), acute lymphoblastic leukemia (*n* = 1), acute non-lymphocytic leukemia (*n* = 1), non-Hodgkin lymphoma (*n* = 1), and obstructive hydrocephalus (*n* = 1). Three cases with alternative diagnoses (2 meningioma, 1 acute non-lymphocytic leukemia) tested positive by both MZN and Xpert MTB/RIF®; they were classified as “definite” TBM cases and received standard anti-TB treatment (isoniazid, rifampicin, pyrazinamide, ethambutol).

The cohort had a median age of 31 years (range 1–80), and the majority (62.3%) of patients were male. All patients were HIV-uninfected. Table 2 provides an overview of relevant clinical, central nervous system imaging, and CSF findings. Among the 316 suspected TBM patients, 38 (12.0%) tested positive with Xpert MTB/RIF®, and 179 (56.7%) with MZN staining; 35 (11.1%) tested positive with both methods.

**Table 2:** **Baseline characteristics of patients evaluated for tuberculous meningitis**.

**Baseline characteristics**	**TBM**			**Not TBM**	
	**Definite *N* = 38 (%)**	**Probable *N* = 66 (%)**	**Possible *N* = 163 (%)**	**IND *N* = 38 (%)**	**NIND *N* = 11 (%)**
Age (years)^*^	25 (7–72)	35 (2–64)	29 (1–80)	34.5 (7–77)	39 (5–57)
Female sex	18 (47)	32 (49)	53 (33)	12 (32)	4 (36)
**HISTORY**					
Fever	24 (63)	54 (82)	113 (69)	26 (68)	3 (27)
Headache	28 (74)	56 (85)	109 (67)	25 (66)	4 (36)
Vomiting	20 (53)	39 (59)	85 (52)	19 (50)	4 (36)
Seizures	2 (5)	10 (15)	27 (17)	4 (11)	1 (9)
Previous TB	2 (5)	3 (5)	2 (1)	0	0
**BMRC GRADING**^a^					
I	15 (40)	27 (41)	85 (52)		
II	5 (13)	7 (11)	36 (22)		
III	18 (47)	32 (49)	42 (26)		
Diagnostic score^*^	14 (10–18)	12 (10–16)^b^	8 (6–11)^c^	8 (3–15)	4 (3–9)
**IMAGING**					
Chest X-ray suggestive of TB	14 (37)	23 (35)	19 (12)	6 (16)	1 (9)
Hydrocephalus	9 (24)	13 (20)	15 (9)	2(5)	1 (9)
Basal meningeal enhancement	9 (24)	8 (12)	16 (10)	5 (13)	0
Infarcts	9 (24)	17 (26)	17 (10)	7 (18)	0
**CSF FINDINGS**					
Total leukocyte count^*^ cells/μl	124 (0–6100)	94 (0–1355)	96 (0–5450)	45 (1–3470)	5 (0–1240)
Neutrophils (%)^*^	23 (0–95)	7 (0–95)	2 (0–94)	2 (0–88)	20 (3–63)
Lymphocytes >50%	24 (63)	52 (79)	114 (70)	30 (79)	8 (73)
Monocytes (%)^*^	10(3–27)	8(0–47)	11 (0–85)	12 (3–51)	21 (8–83)
Glucose < 2.2 mmol/l	15 (40)	38 (58)	36 (22)	13 (34)	1 (9)
Protein >1.0 mg/dl	20 (53)	43 (65)	47 (29)	17 (45)	1 (9)

*TBM, tuberculous meningitis; TB, tuberculosis; IND, infectious neurological disorders; NIND, non-infectious neurological disorders; CSF, Cerebral Spinal Fluid*.

* Median (range);

a BMRC grading—British Medical Research council disease severity grade (British Medical Research Council 1948): stages 1, 2, and 3 were characterized by signs of meningitis with no neurological deficits, focal neurological deficits, and stupor or coma, respectively;

b Scores of 10 and 11 when brain imaging was not available;

c *Scores of 10 and 11 when brain imaging was available*.

Table 3 summarizes the diagnostic accuracy observed with MZN and Xpert MTB/RIF®, using “definite or probable TBM” and “definite, probable or possible TBM” as reference standards. With this approach the diagnostic sensitivity of MZN (88.5%; 95% confidence interval 80.9–93.3 and 61.8%; 95% CI 55.8–67.4, respectively) appeared higher than that of Xpert MTB/RIF® (36.5%; 95% CI 27.9–46.1 and 14.2%; 95% CI 10.6–18.9, respectively), but the specificity compared to “definite or probable TBM” and “definite, probable or possible TBM” was lower (71.4%; 95% CI 57.6–82.2 vs. 100.0%; 95% CI 92.7–100.0). CSF *M. tuberculosis* culture was performed in 201/316 (63.6%) patients; none classified as “Not TBM.” Culture was performed in 104/179 (58.1%) MZN positive samples; 12.5% (13/104) were confirmed as *M. tuberculosis* and no nontuberculous mycobacteria (NTM) were identified. Of the CSF culture positive specimens, 61.5% (8/13) were confirmed as *M. tuberculosis* by Xpert MTB/RIF®. Using MGIT960 culture as the reference standard, MZN had a sensitivity of 100.0% (95% CI 77.2–100.0) compared to 61.5% for Xpert MTB/RIF® (95%CI 35.5–82.3), but the specificity was lower (51.6%; 95%CI 44.5–58.6 vs. 98.9%; 95%CI 96.2–99.7). Using Xpert MTB/RIF® as the reference standard, MZN had a sensitivity of 92.1% (95% CI 79.2–97.3) and specificity of 71.4% (95% CI 57.6–82.2).

**Table 3:** **Diagnostic accuracy of MZN stain compared to “definite or probable TBM,” “definite, probable, or possible TBM,” CSF ***M. tuberculosis*** culture or CSF Xpert MTB/RIF® as reference standards**.

**Reference standard**		**MZN**		**Xpert MTB/RIF**^**®**^	
		**+**	**−**	**+**	**−**
Definite or probable TBM^*^ (*N* = 104)		92	12	38	66
Not TBM (*N* = 49)		14	35	0	49
Definite, probable or possible TBM^*^ (*N* = 267)		165	102	38	229
Not TBM (*N* = 49)		14	35	0	49
CSF *M. tuberculosis* culture positivie^**^ (*N* = 13)		13	0	8	5
CSF *M. tuberculosis* culture negative^**^ (*N* = 188)		91	97	2	186
Xpert MTB/RIF® confirmed (*N* = 38)		35	3	38	0
Not TBM (*N* = 49)		14	35	0	49
**Reference standard**	**Diagnostic performance measure**	**MZN (%)**	**95%CI**	**Xpert MTB/RIF**^®^ **(%)**	**95%CI**
Definite, or probable TBM^*^	Sensitivity	88.5 (92/104)	80.9–93.3	36.5 (38/104)	27.9–46.1
	Specificity	71.4 (35/49)	57.6–82.2	100.0 (49/49)	92.7–100.0
	PPV	86.8 (92/106)	79.0–92.0	100.0 (38/38)	90.8–100.0
	NPV	74.5 (35/47)	60.5–85.0	42.6 (49/115)	34.0–51.7
Definite, probable or possible TBM^*^	Sensitivity	61.8 (165/267)	55.8–67.4	14.2 (38/267)	10.6–18.9
	Specificity	71.4 (35/49)	57.6–82.2	100.0 (49/49)	92.7–100.0
	PPV	92.2 (165/179)	87.3–95.3	100.0 (38/38)	90.8–100.0
	NPV	25.5 (35/137)	19.0–33.5	17.6 (49/278)	13.6–22.5
Positive CSF *M.tuberculosis* culture^**^	Sensitivity	100.0 (13/13)	77.2–100.0	61.5 (8/13)	35.5–82.3
	Specificity	51.6 (97/188)	44.5–58.6	98.9 (186/188)	96.2–99.7
	PPV	12.5 (13/104)	7.5–20.2	80 (8/10)	49.0–94.3
	NPV	100.0 (97/97)	96.2–100.0	97.4 (186/191)	94.0–98.9
Xpert MTB/RIF® confirmed	Sensitivity	92.1 (35/38)	79.2–97.3	N/A	
	Specificity	71.4 (35/49)	57.6–82.2		
	PPV	71.4 (35/49)	57.6–82.2		
	NPV	92.1 (35/38)	79.2–97.3		

*TBM, tuberculosis meningitis; MZN, modified Ziehl-Neelsen staining; CSF, cerebral spinal fluid; PPV, Positive Predictive Value; NPV, Negative Predictive Value; 95%CI-95% confidence interval; N/A, not applicable; M. tuberculosis, Mycobacterium tuberculosis*.

* *Classification according to the consensus uniform research case definition criteria (Marais et al., 2010)*.

** *CSF M. tuberculosis culture was performed in only 201/316 (63.6%) patients; depending on culture availability and the discretion of the treating physician*.

MZN was positive in 14 (28.6%) patients classified as “not TBM.” Table 4 summarizes the clinical profile and outcome of patients in whom a “false positive” MZN test result was recorded. Alternative diagnoses established included cryptococcal (6 cases) and bacterial (6 cases) meningitis, meningioma (1 case) and acute lymphoblastic leukemia (1 case). One patient with cryptococcal meningitis died, but all other patients achieved good outcomes despite only 4/13 (30.7%) receiving anti-tuberculosis treatment. The patient who died had highly elevated intra-cranial pressure and rapidly progressive disease attributed to cryptococcal meningitis. Although the CSF was MZN positive, the Xpert MTB/RIF® test was negative and there was no suggestion of extra-neural TB; no CSF culture was performed and no anti-tuberculosis treatment provided.

**Table 4:** **Clinical profile, treatment and outcome of 14 patients with positive MZN staining of their cerebrospinal fluid classified as “not TBM”**.

**Gender**	**Age (years)**	**Outcome**	**Final diagnosis**	TBM score^*^	TBM treatment^**^	**Other antibiotics**
M	30	Good	Cryptococcal meningitis	9	Y	ceftriaxone, fluconazole
M	7	Good	Cryptococcal meningitis	10	N	ceftriaxone, meropenem, fluconazole
F	49	Good	Meningeoma	9	N	–
M	68	Good	Cryptococcal meningitis	15	Y	fluconazole
F	17	Good	Bacterial meningitis	11	N	ceftriaxone, meropenem, vancomycin
M	54	Good	Bacterial meningitis	9	N	ceftriaxone
M	61	Good	Bacterial meningitis	10	N	ceftriaxone, vancomycin
M	25	Good	Bacterial meningitis	10	N	ceftriaxone
M	5	Good	Acute lymphoblastic leukemia	7	N	ceftriaxone
F	11	Good	Bacterial meningitis	8	N	ceftriaxone
M	39	Good	Cryptococcal meningitis	9	Y	ceftriaxone, fluconazole
M	18	Good	Cryptococcal meningitis	8	Y	ceftriaxone, fluconazole
M	43	Dead	Cryptococcal meningitis	9	N	fluconazole
M	33	Good	Bacterial meningitis	10	N	ceftriaxone, meropenem, ciprofloxacin

MZN, Modified Ziehl-Neelsen; TBM, tuberculous meningitis; M, Male; F, Female; Y, yes; N, no;

* Score according to the consensus uniform research case definition criteria (Marais et al., 2010);

** *Isoniazid, Rifampicin, Pyrazinamide, and Ethambutol; All patients were HIV-uninfected*.

## Discussion

Our findings confirm the high specificity of the Xpert MTB/RIF® assay, but the sensitivity was poor; detecting less than half the patients with “confirmed or probable” TBM. The sensitivity of the Xpert MTB/RIF® assay may have been adversely affected by cold storage and delayed testing, as well as the low sample volumes and low bacterial load in CSF samples. The yield reported in our study is generally comparable to previous TBM studies (Alvarez-Uria et al., 2012; Patel et al., 2013; Bahr et al., 2015), although a study in Uganda reported that the Xpert MTB/RIF® yield more than doubled (72 vs. 28%) using high volume centrifuged CSF specimens (Bahr et al., 2015). Studies reporting higher sensitivity with Xpert MTB/RIF® often enrolled high proportions of HIV-infected patients (Patel et al., 2013; Nhu et al., 2014; Bahr et al., 2015), while no patients were HIV-infected in our study. Although a positive Xpert MTB/RIF® assay result provided a rapid and specific diagnosis, the sensitivity of the Xpert MTB/RIF® assay is not good enough to serve as a “rule out” test (Bahr et al., 2016).

MZN previously demonstrated excellent sensitivity (100%) and good specificity (85.1%) compared to culture-confirmed TBM as the reference standard, suggesting value as a rapid screening test (Feng et al., 2014). However, in the current study MZN results suggested a high number of false positives, with only 12.5% of cultured MZN positive CSF specimens confirmed as *M. tuberculosis* and 28.6% of “not TBM” patients found to be MZN positive. MZN specificity was 71.4% compared to Xpert MTB/RIF® as the reference standard. The fact that a large number of patients who were not “confirmed or probable” TBM cases were MZN positive is a concern, since false positive tests may lead to unnecessary and inappropriate treatment. This is particularly true for the “not TBM” cases in whom an alternative diagnosis was established; most of whom recovered without anti-tuberculosis treatment. Although, many of these patients received short courses of ceftriaxone and/or meropenem, which may have some anti-tuberculosis activity, there is no ancillary evidence to support the positive MZN result and it is highly unlikely that the sub-optimal treatment received would have resulted in cure if these patients had TBM.

The poor specificity observed with MZN could be explained by superior sensitivity compared to the reference standard, but this is unlikely to apply to the clinical reference standard used, given that only “definite and probable TBM” and “not TBM” cases were included in the comparative analysis. Nontuberculous mycobacteria (NTM) may also explain discrepant MZN and Xpert MTB/RIF® results (Menzies and Nahid, 2013), but NTM is an exceedingly rare cause of meningitis (Lawn and Zumla, 2011) and no NTM was identified in any of the samples that grew mycobacteria. Artifacts produced during MZN staining may have been misidentified as mycobacteria, since the technique often distorts the typical appearance of acid-fast bacilli (Chen et al., 2012). Although highly experienced laboratory technicians performed the staining and interpreted the results, the technique may benefit from careful optimization. In general, the positive predictive value of MZN was lower than that of Xpert MTB/RIF®, but the negative predictive value was higher indicating potential value as a preliminary screening test. However, the fact that MZN had a sensitivity of 92.1% compared to Xpert MTB/RIF® limits its use as a rapid screening test in its current format, given fears of potential false negative results.

Study limitations include the fact that *M. tuberculosis* culture was performed on a minority of patients. *M. tuberculosis* culture would have had particular value in potentially false positive MZN cases that tested negative on Xpert MTB/RIF®. The absence of an optimal reference standard that is inclusive of all “true TBM” cases remains problematic and hampers diagnostic test development. Given the current limitations, assessing the diagnostic performance of new tests against multiple reference standards, as done in our study, represents the most rigorous approach. There is a huge need for a triage test with high sensitivity that could rapidly screen potential TBM cases. Despite good sensitivity, the fact that some Xpert MTB/RIF® positive cases were MZN negative is a major concern. Once MZN methodology has been reviewed and optimized it should be re-evaluated in a large prospective study with well characterized patient groups, to specifically explore it value as a rapid, sensitive triage test.

## Conclusion

A positive CSF Xpert MTB/RIF® result provides a rapid and specific TBM diagnosis, but poor sensitivity is a major constraint. MZN is hampered by likely false positives and sub-optimal specificity. Given its increased sensitivity compared to Xpert MTB/RIF®, MZN could be considered as a rapid triage test, but potential false negative results require further evaluation. Strategies to enhance the sensitivity of Xpert MTB/RIF® or improve the diagnostic accuracy of MZN should be explored.

## Author contributions

GZ, YZ were responsible for the design of the study. TW, YY, WD, and YP performed the experiments. TW, LFZ, and JY provided specimens and participated in transportation of strains. TW, LZ participated in medical records and specimen collection. The data were analyzed by TW, YP, and GF, with input from BM, YZ, GZ, GF, and LPZ. TW wrote the first draft of the manuscript. All authors commented on the manuscript revisions and approved the final version.

## Funding

This work was supported by National Key Project (2013ZX10003003) and the National Natural Science Foundation of China (81371334).

### Conflict of interest statement

The authors declare that the research was conducted in the absence of any commercial or financial relationships that could be construed as a potential conflict of interest.

## Footnotes

^1^The tuberculosis control program in Shaanxi province, China. Available online at: http://wenku.baidu.com/view/0ce7d938580216fc700afd6c.html

## References

[B1] Alvarez-Uria G. Azcona J. M. Midde M. Naik P. K. Reddy S. Reddy R. (2012). Rapid diagnosis of pulmonary and extrapulmonary tuberculosis in HIV-infected patients. Comparison of LED fluorescent microscopy and the geneXpert MTB/RIF assay in a district hospital in India. Tuberc. Res. Treat. 2012:932862. 10.1155/2012/932862 22966426 PMC3433122

[B2] Bahr N. C. Marais S. Caws M. Van-Crevel R. Wilkinson R. J. Tyaqi J. S.. (2016). Tuberculous meningitis international research consortium. GeneXpert MTB/Rif to Diagnose Tuberculous Meningitis: perhaps the first test but not the last. Clin. Infect. Dis. 62, 1133–1135. 10.1093/cid/ciw083 26966284 PMC4826457

[B3] Bahr N. C. Tugume L. Rajasingham R. Kiggundu R. Williams D. A. Morawski B.. (2015). Improved diagnostic sensitivity for TB meningitis with Xpert MTB/Rif testing of centrifuged CSF: a prospective study. Int. J. Tuberc. Lung Dis. 19, 1209–1215. 10.5588/ijtld.15.0253 26459535 PMC4768484

[B4] Boehme C. C. Nabeta P. Hillemann D. Nicol M. P. Shenai S. (2010). Rapid molecular detection of tuberculosis and rifampin resistance. N. Engl. J. Med. 363, 1005–1015. 10.1056/NEJMoa0907847 20825313 PMC2947799

[B5] British Medical Research Council (1948). Streptomycin treatment of tuberculous meningitis. BMJ 1, 582–597. 13155481

[B6] Chen P. Shi M. Feng G.-D. Liu J.-Y. Wang B.-J. Shi X.-D.. (2012). A highly efficient Ziehl-Neelsen stain: identifying de novo intracellular *Mycobacterium tuberculosis* and improving detection of extracellular *M. tuberculosis* in cerebrospinal fluid. J. Clin. Microbiol. 50, 1166–1170. 10.1128/JCM.05756-11 22238448 PMC3318527

[B7] Feng G.-D. Shi M. Ma L. Chen P. Wang B.-j. Zhang M.. (2014). Diagnostic accuracy of intracellular *Mycobacterium tuberculosis* detection for tuberculous meningitis. Am. J. Respir. Crit. Care Med. 189, 475–481. 10.1164/rccm.201309-1686OC 24450377 PMC3977721

[B8] Koh W. J. Ko Y. Kim C. K. Park K. S. Lee N. Y. (2012). Rapid diagnosis of tuberculosis and multidrug resistance using a MGIT 960 system. Ann. Lab. Med. 32, 264–269. 10.3343/alm.2012.32.4.264 22779067 PMC3384807

[B9] Krüüner A. Yates M. D. Drobniewski F. A. (2006). Evaluation of MGIT 960-based antimicrobial testing and determination of critical concentrations of first- and second-line antimicrobial drugs with drug-resistant clinical strains of *Mycobacterium tuberculosis*. J. Clin. Microbiol. 44, 811–818. 10.1128/JCM.44.3.811-818.2006 16517859 PMC1393078

[B10] Lawn S. D. Zumla A. I. (2011). Tuberculosis. Lancet 378, 57–72. 10.1016/S0140-6736(10)62173-3 21420161

[B11] Marais S. Thwaites G. Schoeman J. F. Török M. E. Misra U. K. Prasad K.. (2010). Tuberculous meningitis: a uniform case definition for use in clinical research. Lancet Infect. Dis. 10, 803–812. 10.1016/S1473-3099(10)70138-9 20822958

[B12] Menzies D. Nahid P. U. (2013). Update in tuberculosis and nontuberculous mycobacterial disease 2012. Am. J. Respir. Crit. Care Med. 188, 923–927. 10.1164/rccm.201304-0687UP 24127799

[B13] Nhu N. T. Q. Heemskerk D. Thu D. D. A. Chau T. T. H. Mai N. T. H. Nghia H. D. T.. (2014). Evaluation of GeneXpert MTB/RIF for diagnosis of tuberculous meningitis. J. Clin. Microbiol. 52, 226–233. 10.1128/JCM.01834-13 24197880 PMC3911435

[B14] Nicol M. P. Workman L. Isaacs W. Munro J. Black F. Eley B.. (2011). Accuracy of the Xpert MTB/RIF test for the diagnosis of pulmonary tuberculosis in children admitted to hospital in Cape Town, South Africa: a descriptive study. Lancet Infect. Dis. 11, 819–824. 10.1016/S1473-3099(11)70167-0 21764384 PMC4202386

[B15] Patel V. B. Theron G. Lenders L. Matinyena B. Connolly C. Singh R.. (2013). Diagnostic accuracy of quantitative PCR (Xpert MTB/RIF) for tuberculous meningitis in a high burden setting: a prospective study. PLoS Med. 10:e1001536. 10.1371/journal.pmed.1001536 24167451 PMC3805498

[B16] Thwaites G. E. (2013). Advances in the diagnosis and treatment of tuberculous meningitis. Curr. Opin. Neurol. 26, 295–300. 10.1097/WCO.0b013e3283602814 23493162

[B17] Thwaites G. E. van-Toorn R. Schoeman J. (2013). Tuberculous meningitis: more questions, still too few answers. Lancet Neurol. 12, 999–1010. 10.1016/S1474-4422(13)70168-6 23972913

[B18] World Health Organization (2013). Automated Real-Time Nucleic Acid Amplification Technology for Rapid and Simultaneous Detection of Tuberculosis and Rifampicin Resistance: Xpert MTB/RIF Assay for the Diagnosis of Pulmonary and Extrapulmonary TB in Adults and Children: Policy Update. Available online at: www.who.int/tb/laboratory/xpert_launchupdate 25473701

[B19] World Health Organization (2010). Tuberculosis Diagnostics: Automated DNA Test. Available online at: http://www.who.int/tb/features_archive/xpert_factsheet.pdf

[B20] Zhang T. Guo L. Zhang S. Liu W. Chen G. Hui M.. (2011). Improving detection and notification of tuberculosis cases in students in Shaanxi province, China: an intervention study. BMC Public Health 11:147. 10.1186/1471-2458-11-147 21375731 PMC3058021

[B21] Zhao K. Kang W. Liu Q. Li Y. Liu Q. Jiang W.. (2014). Genotypes and transmitted drug resistance among treatment-naive HIV-1-infected patients in a northwestern province, China: trends from 2003 to 2013. PLoS ONE 9:e109821. 10.1371/journal.pone.0109821 25333965 PMC4198111

